# Hearing preservation middle fossa approach for intracanalicular vestibular schwannoma in a NF2 patient

**DOI:** 10.3171/2021.7.FOCVID21121

**Published:** 2021-10-01

**Authors:** Claudio H. F. Vidal, Yoav Hahn, Mariana C. Leal, Kiara Medeiros, Gabriela F. Hazin, Caetano J. Coimbra

**Affiliations:** 1 Department of Neurosurgery, Getúlio Vargas Hospital, Recife, Pernambuco, Brazil;; 2 Skull Base Surgery Center, Baylor University Medical Center, Dallas;; 3 Minimally Invasive Brain Surgery Center, Medical City Hospital, Dallas, Texas;; 4 Department of Surgery, Universidade Federal de Pernambuco, Recife, Pernambuco; and; 5 Faculdade Pernambucana de Saúde, Recife, Pernambuco, Brazil

**Keywords:** acoustic neuroma, middle cranial fossa, neurofibromatosis 2, hearing loss

## Abstract

Hearing preservation is a cornerstone in the management of intracanalicular vestibular schwannomas. This video demonstrates a middle fossa approach to an intracanalicular schwannoma and highlights some technical and anatomical nuances relevant to the procedure. The patient had sustained hearing preservation in the postoperative period. There are potential benefits in favor of the middle fossa when the tumor reaches the fundus of the internal auditory canal, but the surgeon’s individual experience plays a decisive role in the choice of approach.

The video can be found here: https://stream.cadmore.media/r10.3171/2021.7.FOCVID21121

## Transcript

We present a case with surgical video of hearing preservation middle fossa approach for intracanalicular vestibular schwannoma in an NF2 patient.

### 0:32 Clinical Case

This is a 53-year-old female patient with history of familial neurofibromatosis type 2 (NF2). The patient presented to us 5 years ago with bilateral intracanalicular vestibular schwannomas and normal hearing without tinnitus, dizziness, or vertigo. The patient elected to proceed with conservative treatment with surveillance magnetic resonance imaging (MRI) and audiograms. At the preoperative consult, the audiometry was unchanged with a 15-dB pure tone average and 100% speech discrimination bilaterally. There was, however, worsening of the auditory evoked potential with prolonged wave V latency in the left ear.

### 1:17 Imaging Studies

MRI demonstrated bilateral enhancing tumors extending to the fundus of the internal auditory canal (IAC). The left-sided tumor measured 1.0 × 0.6 cm as compared to previous imaging, 0.8 × 0.4 cm, and the right-sided tumor measured 1.2 × 0.5 cm as compared to 0.8 × 0.5 cm on MRI 5 years prior. FIESTA MRI sequence clearly showed the cranial nerves VII and VIII in the cerebellopontine angle (CPA).

### 1:54 Case Management Decisions

Surgical intervention was recommended based on the progression of the tumor size and worsening of the auditory evoked potentials. The side of the surgery was chosen based on the changes of the auditory evoked potentials on the left. We, however, understand that some authors may have preferred to operate on the side with better evoked potential parameters.^1^ The middle fossa hearing preservation approach was recommended based on the size of the tumor, hearing status, intracanalicular location of the tumor with extension to the fundus of the IAC, and the senior author’s published experience.^2–4^ We, however, understand that some authors would have recommended the retrosigmoid transmeatal approach.^5^ Radiosurgery is an option in intracanalicular schwannomas to control tumor growth, but functional hearing is preserved in only 44% to 63% of cases.^6^ These hearing preservation percentages are lower than the results of the senior author for this type of tumor (73.3% of hearing preservation rate).^2^

### 2:52 Patient Positioning

The position of the head is important. We recommend 30° head elevation for brain relaxation. Rotation of the head should not be more than 30° to the opposite side to allow a favorable vantage point in line with the long axis of the IAC and the oblique oriented superior semicircular canal. We think that further rotation of the head rotates the long axis of the IAC away from the craniotomy line of view and causes the arcuate eminence to hinder access to the IAC.

### 3:15 Craniotomy

A temporal craniotomy is created, measuring about 5.0 × 6.0 cm. We center the craniotomy two-thirds anterior to the external ear canal and one-third posterior to it. The craniotomy is flattened with the middle fossa skull base. We think that the anterior and low location of the craniotomy is important to allow access to the V3 branch of the trigeminal nerve and to allow anterior-to-posterior unobstructed access and visualization of the IAC, which is anterior to the obliquely oriented superior semicircular canal (SSC).

### 3:51 Dural Elevation

Elevation of the temporal lobe dura away from the middle fossa skull base starts with coagulation and section of the middle meningeal artery at the foramen spinosum. Next, the dura is elevated from the V3 branch of the trigeminal nerve just anterior to the artery. The dissection progresses posteriorly with peeling of the dura away from the gasserian ganglion dura propria. The greater superficial petrosal nerve (GSPN) and the arcuate eminence are identified. We believe that this wide dural elevation is necessary to allow ample exposure of the superior surface of the petrous bone without undue focal retraction of the temporal lobe brain parenchyma.^7^ Fixed mechanical brain retractors are not used. Brain spatulas are used sporadically to protect the temporal lobe dura from the shaft of the drill.

### 4:30 Arcuate Eminence

The arcuate eminence is clearly identified. The SSC is blue lined. This allows maximization of the posterior extension of the dural exposure. The posterior limit of the IAC is identified.

### 4:45 Petrous Apex Drilling

Drilling starts at the petrous apex. We think that this is a safe spot to start the bone removal.

### 4:52 Geniculate Ganglion

The geniculate ganglion is exposed and identified with the nerve integrity monitor probe. The course of the facial nerve in the facial nerve canal is identified and topographic orientation of the cochlea is then possible.

### 5:06 Internal Auditory Canal

This allows safer further drilling of the petrous apex, which then progresses posteriorly to find the anterior limits of the IAC. Removal of bone over the IAC allows progressive definition of the borders of the IAC. Bone is then removed from the petrous ridge, superior to the porus acusticus, to allow access to the posterior fossa CPA dura.

### 5:39 Facial Nerve Canal

The bone removal progresses toward the fundus of the IAC and the facial nerve canal. The nerve integrity probe is used for identification of the facial nerve at its labyrinthine segment in the facial nerve canal. The roof of the facial nerve canal is opened. This allows further topographic orientation of the location of the cochlea anterior to the facial canal.

### 5:52 Fundus of the IAC

Prior to further drilling at the dangerous area of the fundus of the IAC, the important anatomical structures should be clearly identified. This includes the arcuate eminence, the facial nerve canal, the geniculate ganglion, and the cochlea.

### 6:07 Dural Exposure at the Fundus

The bone removal then continues at the fundus of the IAC. Exposure of the dura all the way to the end of the IAC is essential for accessing the lateral limit of the tumor, permitting safe resection. Thorough exposure of this area, however, comes close to the cochlea, facial nerve canal, and the ampulla of the SSC. Careful attention is necessary to avoid injury to these structures. A 1.5 diamond drill bit is used at this stage, with plenty of irrigation.

### 6:30 Dural Opening

The dura is widely opened starting posteriorly to avoid the facial nerve anterior trajectory in the IAC. The dural opening continues to include all the dura of the IAC, porus, petrous ridge, and petrous apex. This wide dural opening allows visualization of the entirety of the tumor and the anatomy of the CPA. The dura at the fundus is not opened at this initial stage.

### 6:49 Initial Tumor Debulking

Debulking of the tumor starts posteriorly to avoid facial nerve injury and continues at the more superficial part of the tumor.

### 6:56 Vestibulocochlear Nerve

The proximal vestibulocochlear nerve is identified posterior and under the tumor at the CPA. Tumor debulking continues after clear identification of the vestibulocochlear nerve.

### 7:12 Facial Nerve

In this case, the facial nerve is encountered at the IAC anterior and under the tumor as opposed to the more common superficial location of the nerve.^3,4^ The tumor is peeled away from the facial nerve.

### 7:32 Facial Nerve Dissection

The origin of the tumor is clearly seen at the superior vestibular nerve. Tumor dissection away from the more proximal aspect of the facial nerve continues until the nerve is completely separated from the capsule of the tumor. Note the good plane of dissection between the nerve and the tumor.

### 7:54 Superior Vestibular Nerve

The origin of the tumor at the superior vestibular nerve is cut, and the cochlear nerve is identified posterior to the facial nerve and under the tumor. Notice the cochlear nerve more posterior in the depth of the exposure and the origin of the tumor at the superior vestibular nerve in a more superficial location.

### 8:16 Tumor Removal

The tumor is removed and passed out of the field. After resection of the tumor, the cochlear nerve and the facial nerve can be seen in the tumor bed cavity.

### 8:27 Final View

The proximal facial nerve is stimulated with the facial nerve integrity probe at 0.02 mAmp.

### 8:37 Closure

All petrous bone air cells are sealed with bone wax, as shown on the photo. The dural defect is obliterated with temporal muscle autograft. The craniotomy in this case was reconstructed with a titanium mesh, but more commonly we use the osteoplastic bone flap. The soft tissue is closed by layers.

### 8:44 Postoperative Course

At 18 months’ follow-up, the patient has sustained hearing preservation without tinnitus, vertigo, or balance difficulties.

### 8:52 Postoperative Audiogram

Audiogram at 11 months postoperative is unchanged from preoperative audiogram with a 15-dB pure tone average and 100% speech discrimination.

### 9:03 Postoperative Computed Tomography

Postoperative CT shows extensive resection of the petrous bone around the IAC and petrous apex. A titanium mesh was used for bone reconstruction.

### 9:12 Postoperative Postcontrast MRI

Postoperative postcontrast MRI at 12 months shows no evidence of residual or recurrent tumor.

### 9:20 Postoperative FIESTA MRI

Postoperative FIESTA sequence MRI at 12 months shows intact facial nerve and cochlear nerve from CPA to the cochlea.

### 9:30 Conclusion

We present a case and surgical video of middle fossa hearing preservation approach as a viable option for treatment of intracanalicular vestibular schwannoma in NF2 patients. The approach, however, is technically demanding, and expertise with the surgical technique is required to achieve successful outcomes. We share technical pearls learned over 20 years of experience with middle fossa approach for acoustic neuroma.

## Disclosures

The authors report no conflict of interest concerning the materials or methods used in this study or the findings specified in this publication.

## Author Contributions

Primary surgeon: Coimbra. Assistant surgeon: Hahn, Leal. Editing and drafting the video and abstract: Vidal, Hahn, Hazin. Critically revising the work: Vidal, Hahn, Leal, Medeiros. Reviewed submitted version of the work: Vidal. Approved the final version of the work on behalf of all authors: Vidal. Supervision: Vidal, Medeiros.
